# Sacral Pressure Ulcer

**Published:** 2013-01-23

**Authors:** Paul J. Therattil, Craig Pastor, Mark S. Granick

**Affiliations:** Department of Surgery, Division of Plastic Surgery, New Jersey Medical School, University of Medicine and Dentistry of New Jersey, Newark

## DESCRIPTION

The patient is a 53-year-old man with paraplegia, who has developed a sacral pressure ulcer after being bedridden in the supine position. The patient presented for debridement and reconstruction of the ulcer.

## QUESTIONS

1. **What are the specific risk factors for sacral pressure ulcers?**
2. **What is a risk assessment tool?**
3. **What options exist for debridement?**
4. **What options exist for reconstruction?**

## DISCUSSION

Pressure (decubitus) ulcers are wounds that form as a direct result of pressure over a bony prominence. Seventy-five percent of these injuries occur around the pelvic girdle, most often at the ischium, greater trochanter, and sacrum. Tissue ischemia occurs at these sites when external pressure exceeds capillary bed pressure, which ranges from 12 to 32 mm Hg depending on the medical status of the patient. Apart from ischemia, other factors that prevent normal healing from occurring include poor nutrition, infection, edema, persistent moisture, fecal and urinary soiling, and shearing forces. Specific risk factors for sacral pressure ulcers include lying in the supine position and fecal incontinence.

The Braden Risk Assessment Scale can be utilized to assess a patient's risk of developing a pressure ulcer. The scale assesses levels of sensory perception, moisture, activity, mobility, nutrition, and friction. All categories are rated on a scale from 1 to 4, except the friction criteria, which is on a scale to 3. The closer the score is to the maximum of 23, the lower the risk of developing a pressure sore. Adults with scores lower than 18 are considered high risk. Other scales, such as the Norton Scale, are also available.

The advantages of early surgical intervention include reducing spread of infection, improving quality of life, facilitating rehabilitation, and decreasing mortality. Prior to surgery, it is necessary to eliminate contributing factors preventing wound healing. The approach is multifaceted and involves optimizing nutrition, controlling infection, improving the overall medical condition, and eliminating sources of external pressure.

Initial surgical management includes debridement. This serves multiple purposes including removing infected, necrotic, and desiccated tissue; providing tissue for culture and biopsy; and preparing the wound for future reconstruction. Debridement options include mechanical, biological such as maggot therapy, enzymatic, and surgical methods. Surgical options include wide excision (centripetal) or centrifugal using a tangential debridement device like the Versajet (Smith and Nephew, Hull, United Kingdom).

Reconstruction can be performed immediately after debridement or may be delayed until other contributing factors are eliminated. Options low on the reconstructive ladder, such as primary closure and skin grafting, are less appropriate methods for closing a sacral wound. Primary closure of these wounds is avoided because of the high rate of recurrence with closure over the bony prominence. Skin grafting has a low success rate and is often precluded because of the frequent presence of a bony wound bed and the absence of adequate soft tissue bulk. Flaps are usually more efficacious methods of reconstruction as they allow vascularized tissue to facilitate wound healing and provide padding to redistribute pressure over the area. Common flap options for sacral pressure ulcers include musculocutaneous and fasciocutaneous flaps. Numerous random skin flaps and free flaps have also been described. The gluteus maximus muscle is used often for musculocutaneous flaps and may be designed as a rotation flap, advancement island flap, or split flap. Thoracolumbar, posterior thigh, extended tensor fascia lata, and total thigh flaps may be used when alternatives are necessary. Fasciocutaneous flaps, such as the superior gluteal artery perforator flap, have been proposed by some to have advantages over musculocutaneous flaps in the treatment of sacral pressure ulcers. Some of the considerations when choosing the appropriate flap include size and recurrence of the pressure ulcer, the ambulatory status of the patient, and prior surgeries performed to reconstruct the defect. Our preferred method of reconstruction is a gluteus maximus musculocutaneous V-Y patterned flap. A second option is a flap from the posterior thigh.

### Postoperative commentary

This patient had a successful gluteus maximus musculocutaneous flap. Postoperative care was directed toward prevention of recurrence, including an air-fluidized (high air-loss) mattress immediately after operation. This was followed by pressure reduction, nutrition optimization, and a turning protocol.

## Figures and Tables

**Figure 1:**
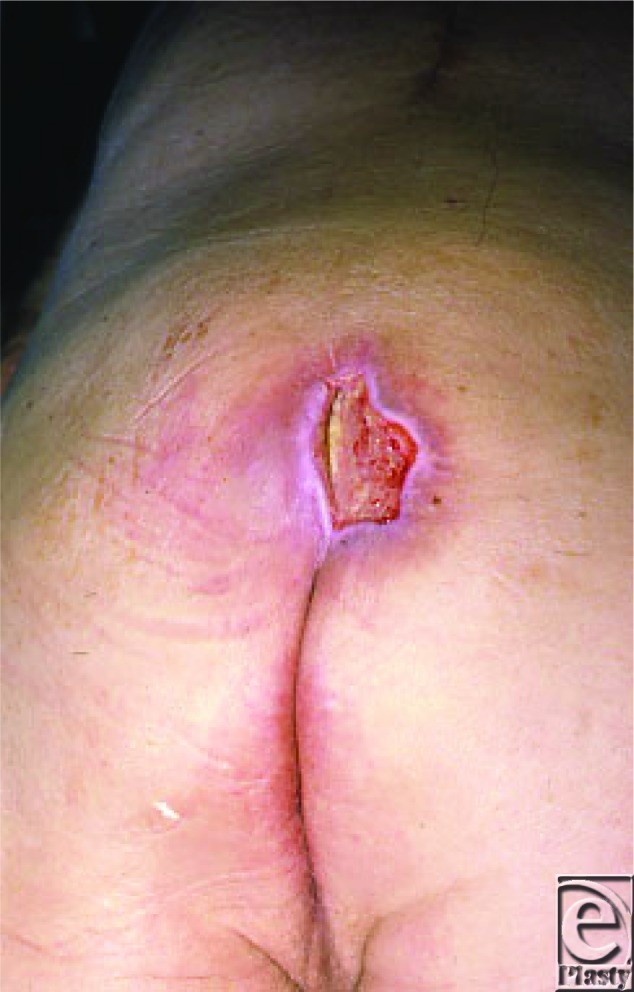
Sacral ulcer, Stage IV.

**Figure 2:**
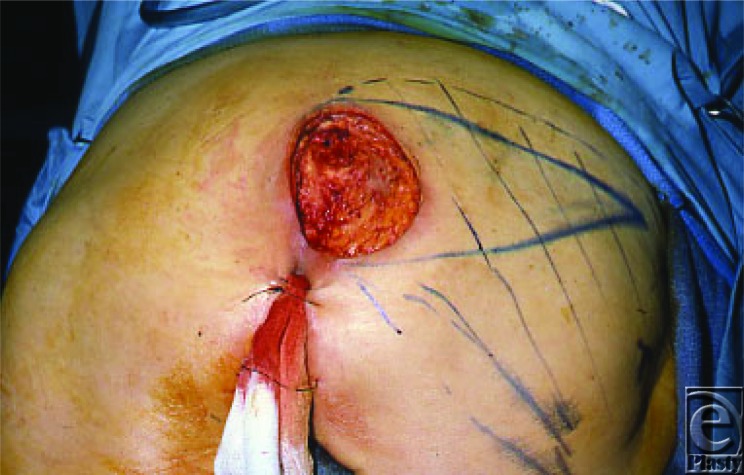
Debrided ulcer.

**Figure 3:**
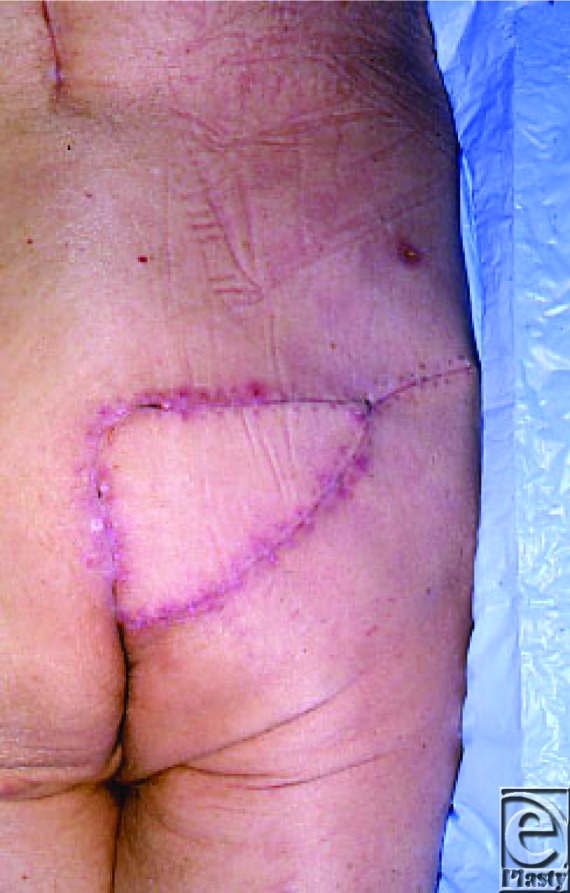
Healed wound following Gluteus Maximus myocutaneous flap repair.
